# The Nordic Maintenance Care Program: Maintenance care – what happens during the consultation? Observations and patient questionnaires

**DOI:** 10.1186/2045-709X-20-25

**Published:** 2012-08-10

**Authors:** Marita Bringsli, Aurora Berntzen, Dorthe Brandborg Olsen, Lise Hestbæk, Charlotte Leboeuf-Yde

**Affiliations:** 1 Private practice, Arna Kiropraktorsenter, P.b. 215 Indre Arna, Bergen, 5888, Norway; 2 Private practice, Øveråsklinikken, Olav Tryggvasons gt 2B, Trondheim, 7011, Norway; 3 Institute of Clinical Biomechanics, University of Southern Denmark, Odense, Denmark; 4 Nordic Institute of Chiropractic and Clinical Biomechanics, Odense, Denmark; 5 Institute of Regional Health Services Research, University of Southern Denmark, Odense, Denmark; 6 Research Department, Spine Center Centre of Southern Denmark, Middelfart, Denmark

**Keywords:** Chiropractic, Maintenance care, Back pain, Consultation

## Abstract

**Background:**

Because maintenance care (MC) is frequently used by chiropractors in the management of patients with back pain, it is necessary to define the rationale, frequency and indications for MC consultations, and the contents of such consultations. The objectives of the two studies described in this article are: i) to determine the typical spacing between visits for MC patients and to compare MC and non-MC patients, ii) to describe the content of the MC consultation and to compare MC and non-MC patients and iii) to investigate the purposes of the MC program.

**Method:**

In two studies, chiropractors who accepted the MC paradigm were invited to assist with the data collection. In study 1, patients seen by seven different chiropractors were observed by two chiropractic students. They noted the contents of the observed consultations. In study 2, ten chiropractors invited their MC patients to participate in an anonymous survey. Participants filled in a one page questionnaire containing questions on their view of the purposes and contents of their MC consultations. In addition, information was obtained on the duration between appointments in both studies.

**Results:**

There were 178 valid records in study 1, and in study 2 the number of questionnaires received was 373. The time interval between MC visits was close to nine weeks and for non-MC consultations it was two weeks.

The content of the consultations in study 1 was similar for MC and non-MC patients with treatment being the most time-consuming element followed by history taking/examination. MC consultations were slightly shorter than non-MC consultations.

In study 2, the most common activities reported to have taken place were history taking and manipulative therapy. The most commonly reported purposes were to prevent recurring problems, to maintain best possible function and /or to stay as pain free as possible.

**Conclusions:**

The results from these two studies indicate that MC consultations commonly take place with around two months intervals, and that history taking, examination and treatment are as important components in MC as in non-MC consultations. Further, the results demonstrate that most patients consider the goal to be secondary or tertiary prevention.

## Background

### Present level of evidence

Maintenance care (MC) is a concept well known among chiropractors, although it is poorly defined and rarely studied. A literature review published in 1996 concluded that there was no scientific evidence to support the claim that MC improves health status and recommended that a series of research actions should be taken [1].

A new review carried out in 2008 revealed, among other things, that not only was there still no evidence-based definition of MC but also that although many chiropractors believe in its usefulness, it seems to be less well accepted by patients [2]. This statement was based on one study, in which it was stated that 79% of patients were recommended for MC by their chiropractors but that only 34% of patients elected to receive these services [3]. Nevertheless, MC programs seem to be relatively common with more than one fifth of visits to Scandinavian chiropractors being MC visits [4,5].

Since then, the effect of MC has been investigated with varying results in one pilot study and two randomized controlled studies. The pilot study included low back pain patients [6] and the two full-scale studies included chronic non-specific neck pain and chronic non-specific low back pain, respectively [7,8].

In the three studies described above, patients were included based on neck or back pain alone, but apparently without taking into account the underlying rationale for MC. Therefore, there is still a need to describe the use of MC in everyday chiropractic practice and to use this knowledge to design appropriate clinical trials of the effect of MC. It is our opinion that it is necessary first to define the inclusion criteria for this type of treatment and to describe best practice or, at least, most common practice in relation to MC to ensure that clinical studies on the effect of MC are carried out on relevant patient populations and in a manner that best reflects clinical reality.

### Spacing between maintenance care consultations

At the time of the second literature review on MC [2], the frequency of and spacing between treatments had been described only in a case-report [9]. More recently though, a study was performed in which chiropractic students observed patients in a number of chiropractic clinics and filled in a questionnaire in relation to MC (yes/no), time for last visit and time for next visit, making it possible to determine the most commonly used interval between appointments for non-MC patients as compared to MC patients [5]. That study revealed a clear distinction between these two types of patient categories in relation to the time between visits. Weekly appointments were most commonly found for the first group, whereas the most commonly selected interval for the next visit was between 1 and 3 months for patients defined by their chiropractor as being MC patients. This information needs to be substantiated in other study populations for two main reasons. First, in a future RCT, it would be necessary to know what the “usual” MC program looks like, in order to conduct the study in a manner which resembles that of real life. Second, in cases of malpractice and when clinicians are reviewed by licensing boards and health authorities, it would be useful to have a benchmark of what can be considered the norm.

### Contents of maintenance care consultations

The contents of MC consultations have only been reported in three studies [3,10,11] and in one case-report [9]. Rupert [3] asked a number of North-American chiropractors to describe the therapeutic components of MC, which were found to be adjustments/spinal manipulation, exercises, proper eating habits, patient education, and vitamin supplementation. This was similar to findings from a study on older patients by the same author [11], and to the findings in another study of Australian chiropractors [10]. However, in none of these studies was this issue considered from the patient’s perspective, nor did the data seem to originate from patient files but were based on the chiropractors’ self-reported activities. We therefore do not know if this is an accurate description of what happens during the MC consultations. To have the patients’ side of the story would be helpful as would an objective account of what actually takes place. In addition, it would be necessary to see if an MC consultation differs from that of a non-MC consultation. One could, for example, expect that continued MC would demand less acute care of the back problem and more advice on life-style and ergonomics at work and during leisure time.

### Rationale for maintenance care

The rationale for MC among chiropractors varies from the concept of secondary and tertiary prevention of musculoskeletal disorders to more all-encompassing approaches such as treatment of subluxations and maintaining and optimizing health in general [3,10-12]. In relation to low back pain, data relating to the 12-months follow-up of chiropractic patients with persistent pain at base-line, who participated in a practice-based outcome study, indicated that MC was provided mainly as tertiary prevention [13]. Primary prevention is the term used when attempting to prevent a disease from developing in the first place. Secondary prevention refers to the prevention of recurrences, whereas tertiary prevention consists of treatment to prevent deterioration and to maintain as good a function as possible in patients who cannot become free of disease or symptoms. Nevertheless, a later study showed that chiropractors firmly believe that they can prevent relapses of back pain, i.e. that they are capable of providing secondary prevention [14]. Whether patients share this opinion is not known. We therefore do not know the reasons why some patients opt for the MC strategy and to what degree, those who do, find it useful.

### Objectives

Two studies were undertaken in order to shed some light on these issues. Study 1 is an observational study of the contents of both MC and non-MC consultations and the spacing between visits. MC and non-MC consultations will be compared. Study 2 is a questionnaire survey of MC patients, investigating the contents of consultations and the spacing between them as well as the purpose of and satisfaction with MC treatment. Where relevant, results from the two studies will be brought together.

## Method

### Study 1. Observation of chiropractic consultations

#### Observers

The two observers were 5^th^ year students of clinical biomechanics (chiropractic) at the University of Southern Denmark with clinical experience from a teaching secondary care clinic and from observational courses in primary care.

#### Participating chiropractors

Eleven chiropractors were approached and 10 agreed to participate in the study. The eleven chiropractors were chosen to represent the three major chiropractic educational institutions relevant in Denmark and an equal distribution between men and women. They were also required to have at least three years of clinical experience. However two of them stated that they did not see MC-patients and for one chiropractor it was not possible to coordinate the schedule with the observers. Thus, seven chiropractors working in four different clinics were chosen to participate and accepted that two chiropractic students would observe their consultations. These seven did represent the three major chiropractic institutions and both genders (two were graduates from the Anglo-European College of Chiropractic, two from Palmer College of Chiropractic, and three from the University of Southern Denmark; four men and three women).

#### Data collection

The students recorded information on all patients seen by a specific chiropractor on a specific week days (e.g. Monday). This day was selected by the chiropractor as a day when MC patients were likely to be present in the clinic. This was done twice for each chiropractor. The first day, data were collected by one of the students and the second time by the other student. The observers did not interfere with the consultations and attempted to be as non-intrusive as possible, so as not to influence the course of the consultation. Only patients who returned for follow-up treatment were included in the study, meaning that new patients or previous patients with a new problem were not eligible for participation in the study.

The observers used a pre-printed observation form to record the types of services rendered and the time spent on each component. On this form the observers registered the number of consecutive slots of 30 seconds spent on each of four predefined content categories: (i) patient history/examination, (ii) treatment, (iii) advice, and (iv) conversation unrelated to the patient’s health. The type of advice and the type of treatment were also noted. In addition, date of observation, date of last visit, date for next visit, gender, age, and primary complaint were recorded. Before the consultation, the chiropractor was asked by the observer to categorize the patient as either an MC patient or a non-MC patient, which was registered on the form. MC was defined as secondary or tertiary prevention. The user friendliness of the observation form had been tested by the students with the help of a chiropractor in a clinic that was not otherwise involved in the study. The form was found to be adequate for its purposes.

Patients’ participation in the study was voluntary, the study did not influence treatment, data were collected anonymously and no sensitive data were collected. The students collected their data under full confidentiality. Therefore, according to Danish law, no approval was necessary from the local ethics committee [15].

#### Data analysis

Data were entered in Epidata, checked for quality and transferred to Stata 10.0 for descriptive analysis. Comparisons were made between MC and non-MC patients and, where appropriate, estimates have been surrounded by 95% confidence intervals. Non-overlapping intervals are considered to indicate statistically significant differences between estimates. There was not enough information available to stratify results on specific chiropractor characteristics. It was not possible to perform a comparison between chiropractors due to the small sample.

### Study 2. Questionnaire survey of patients receiving maintenance care

#### Participants

Eleven chiropractors who had committed themselves to collaboration and research regarding MC constituted a back-up group for the Nordic Maintenance Care Program. They were contacted, as it was certain that they would have access to patients who had received MC. Ten of these chiropractors participated in the data collection of the present study. Consecutive patients, who by these chiropractors were categorized as receiving MC, were invited to participate in the study.

#### Data collection

During a limited period these chiropractors would hand out a one-page questionnaire to all their MC patients. On one side of the questionnaire there was an invitation to participate in the survey and an explanation of how to go about it as well as the reasons for the study. On the other side of the letter there were 7 questions. These were: gender (male/female), age in categories of 10 years each, number of weeks since the last chiropractic visit (open question), number of weeks till the next visit (open question), a list of items that may have been included in the visit of that day (history relating to spinal problems, history relating to other problems, discussion of personal problems, examination, manipulative treatment, soft tissue treatment, instructions to exercises, advice on daily living, and small talk), a list of objectives for the MC (prevention of recurrences, remain as pain free as possible, and prevent disease in general), and a question on whether these objectives had been attained (yes to a high degree/to some degree/hardly).

Patients handed back their questionnaire to the clinic secretary, who forwarded it to the research office at the Nordic Institute for Chiropractic and Clinical Biomechanics, without the treating chiropractor having access to the data. The patient had been informed in the explanatory letter about this procedure. No information is available on the number or type of patients who did not have time, did not want or could not participate in the survey. However, any non-participation is likely to be due to administrative problems or time constraints rather than a wilful selection bias.

The questionnaire was anonymous, participation voluntary, questionnaire responses had no influence on the chiropractic treatment, and no sensitive data were collected. Therefore, again, no approval was necessary from the local ethics committee [15].

#### Data analysis

Data were entered in Epidata, checked for quality and transferred to Stata 10.0 for descriptive analysis.

## Results

### Study1. Observation of chiropractic consultations

#### Description of study sample

All patients who were approached for the study accepted to have their consultation observed. Of 188 observed consultations, 9 were excluded from analysis because of faulty inclusion criteria and one because the form was incorrectly filled out. This left 178 patients for the analysis.

Female patients made up 67% of the MC patients and 65% of the non-MC patients. There were 8% under the age of 30, 53% were from 30 to 55 years of age and 39% older than 55. The proportion of patients receiving MC was 41% and increased with age (21%, 35% and 54% for the three age groups, respectively).

#### Spacing between visits for MC patients and non-MC patients

The mean interval between visits was 8.6 weeks for MC patients (95% CI: 7.6-9.6) ranging from <1 to 113, and 2.4 weeks for non-MC patients (95% CI: 2.0-2.8) with a range of <1 to 50. The most common interval between visits for MC patients was three months, and for non-MC patients it was one week. Twelve (16%) of the MC patients and 11 (10%) of the non-MC patients did not receive a new appointment, but were told to call when needed and four (4%) of the non-MC patients concluded their course of treatment on that particular appointment.

#### Areas of treatment

The complaints for both types of patients were predominantly spinal with lumbar problems being most common. It was noted that MC patients received treatment, on average, in 2.5 areas per patient whereas non-MC patients only received treatment in 1.7 areas per patient. The whole spine was more commonly treated in the MC group (with a statistically significant difference for both the cervical and the lumbar spine), which could indicate more focus on the whole spine for MC patients than for non-MC patients.

#### The contents of the MC and the non-MC consultation and the time spent on each component

**Figure 1:**
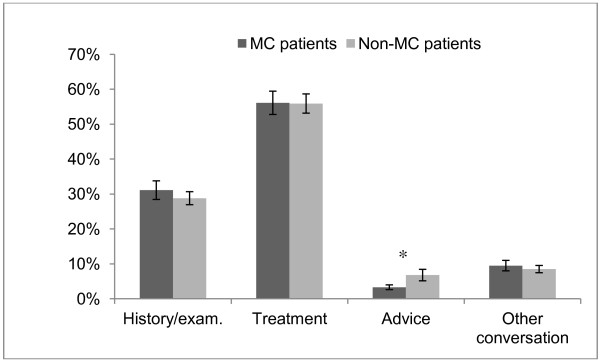
**The proportion of time spent on each of the four components of the consultation.** *: statistically significant difference (p<0.05).

The mean time spent on an MC consultation was 9.2 minutes (95% CI: 8.4-10.0) and for a non-MC consultation it was 11.1 minutes (95% CI: 10.6-11.6). The difference was barely statistically significant but there were considerable variations between chiropractors. The contents and the time distribution were similar between the two types of consultation. Treatment was the most time consuming element for both groups, followed by history taking/examination. Only the time spent on advice differed somewhat between MC and non-MC consultations with more time spent on non-MC patients. The proportion of time spent on each component is shown in Figure 1.

Almost all patients in both groups received spinal manipulation (96% for MC patients and 92% for non-MC patients). The other most commonly used types of therapy were trigger point therapy (64% and 77%, respectively) and massage (38% and 54%, respectively). The differences between MC and non-MC patients were not statistically significant.

In total, 26% of MC patients and 34% of non-MC patients received advice as noted on the observation form. Exercise was most often the issue, followed by ‘other’ and diet, with circumstances at work being mentioned for only 5 non-MC patients and no MC patients.

#### Difference between MC and non-MC consultations

Overall, the MC consultations were shorter than the non-MC consultation, but the contents were similar with the only difference being the time spent on advice and the fact that no MC patients received advice about work. However, the results regarding advice should be interpreted tentatively due to the few participants who did receive advice.

### Study 2. Questionnaire survey of patients receiving maintenance care

#### Description of study sample

The ten participating chiropractors provided a mean number of 37 MC patients, with a range of 15 to 75. The total number of questionnaires returned from patients was 373. It is not possible to report the number of MC patients who passed through the clinics during the time of the study and therefore a response rate cannot be calculated. There was no information on their types of complaints.

Female patients made up 61% of the respondents. The two largest age groups were 40–49 and 50–59, accounting for 54% of the participants. Those younger than 30 made up 5% and 30% were 60 or older. Only two participants were younger than 20 whereas 38 were 70 or more.

#### Spacing between visits for MC patients

The mean time between visits was 9.3 weeks (95% CI: 8.6-10.0) with a range of <1 to 52. The vast majority of participants reported that their last visit took place within 12 weeks (82%). The two most commonly reported specific time intervals since the last consultation were 12 weeks (19%) and 4 weeks (17%).

#### The contents of the MC consultation

Almost all patients (92%) reported that they had discussed with their chiropractor how their back/neck had felt since the last visit. Almost as many, 88%, reported to have received manipulative treatment whereas only 66% thought that they had been examined. Fifty-one percent received some sort of manual muscular treatment.

Other relatively common activities were discussion of other health problems than those of the back/neck and discussion of other personal circumstances, reported by 23% and 17%, respectively.

Instructions on how to perform exercises or reminders to do so were reported by 20% and 10% of patients, respectively, and 20% recalled that they had discussed positions at work and/or other life style factors (for example nutrition, physical activities, or smoking). Only a very small minority reported definite “small-talk” (<1%).

#### The rationale for the MC program and the satisfaction with this

More than three out of four patients considered the purpose of their MC program to be to prevent recurrences (78%) and for almost two thirds the goal was to remain as pain free as possible (63%). Only 17% thought that a reason was to prevent disease in general and 2% reported some “other” (unspecified) reason.

In all, 82% stated that they achieved their goal(s) with MC to a high degree and 17% to some degree.

## Discussion

The two studies describe timing and content of maintenance consultations from two different angles. Study 1 is an attempt at objective observations and study 2 reflects the patients’ perspective. The MC patients from the two studies were comparable with regard to age and gender.

### Spacing between visits for MC patients

The time between MC visits was approximately the same in the two study populations. However, there was a clear distinction in the timing of visits between MC and non-MC patients with visits being scheduled considerably closer for non-MC patients. This corresponds well with previous findings from two Scandinavian studies [4,5], and probably reflect real life, as the values have good face value.

### The contents of the MC consultation

There seems to be a good correlation between the observed elements of the consultation and the patients’ reporting of contents with history taking, examination, manipulation and soft tissue treatment being the predominant elements with most time spent on treatment. These findings are in line with the survey of North American chiropractors [3], where spinal adjustment/manipulation was the most frequent therapeutic component.

### Rationale for the MC and the satisfaction with this

Most patients considered the purpose of their MC to be secondary or tertiary prevention or a combination of the two. This indicates that patients share the chiropractors’ belief that MC can prevent recurrences of pain [14]. It is not known whether this is due to serious consideration on the patients’ part or if it simply reflects that the patients, who comply with a MC programme, trust their chiropractors and adapt their views. It would therefore be interesting to interview patients who decline to be enrolled in a MC programme to get an understanding of why MC is attractive to some but not to others.

Almost all MC patients (99%) felt that they achieved their goal by receiving MC. This is hardly surprising, since patients who are not satisfied must be expected to have dropped out of the programme. To obtain a differentiated view of the patients’ satisfaction, the patients who leave the programme must be included as well. However, the result does show that the patients do not simply comply out of respect for their chiropractor, but actually are content with their treatment.

### Difference between MC and non-MC patients

The consultations were slightly longer for non-MC patients than for MC patients, but there were only very small differences in the composition of the consultations, indicating that active treatment is as common in MC patients as in non-MC patients. The chiropractors spent more time on advice for the non-MC patients, which might reflect that most of the advice is given in the beginning of a treatment course. Although 30% of the patients in study 2 reported to have received advice, this does not have to take up much time if it is merely repetition of previously given advice. Very little time was spent on “other conversation”, i.e. “small talk”. It could be anticipated that the MC patients would spend more time on small talk as they get to know the chiropractor better and there may be less to report with regard to their complaint. The results from study 1 indicate that this is not the case. However, it is possible that the presence of an unknown observer limits the amount of personal conversation and thus the results might not be accurate and should be interpreted cautiously on this account.

### Methodological considerations

The main strengths of these two studies are that they complement each other. Unlike so many other studies on this topic, data are not given by chiropractors who estimate their own patient profiles and clinical procedures. In one of the studies, observers took detailed notes of what was going on. The risk of classification error in the time recording is minimal because the observers are fifth year chiropractic students with clinical experience, the registration was very simple and the observation sheet was pretested in chiropractic practice. In the other study the patients themselves provided the data. The risk of obsequiousness bias was small because the questionnaires were handled by the clinic secretary who forwarded them to the research institute, thus the information was not available for the treating chiropractor.

There are two main weaknesses of the study: 1) The small study sample in the observational study could easily affect the comparison of time spent on advice between MC- and non-MC patients if a few non-MC patients have their x-rays explained, and 2) The presence of observers might hinder the free conversation and thereby decrease the amount of time spent on small-talk. Furthermore, there was some variation among the chiropractors and the seven included chiropractors might not be representative of all the Danish chiropractors, although they were chosen to represent the three major chiropractic educational institutions and represent both genders. Finally, it is a problem that nothing is known about the patients who decline MC, and it is not known if these results can be reproduced in other cultural settings.

## Conclusion

Taking weaknesses and strengths of these studies into account, it seems reasonable to conclude that:

1. Patients have the same expectations to MC as chiropractors and they consider MC to be synonymous to secondary or tertiary prevention of back problems.

2. There are no major differences in contents between MC and non MC consultations. History taking, examination and treatment are equally important components in both.

3. MC visits are more widely spaced than non-MC visits, typically at about 2 months interval.

## Competing interests

The authors declare that they have no competing interests.

## Authors’ contributions

MB, AB, LH and CL-Y contributed to conception and design of study 1, MB and AB contributed to acquisition of data, analysis and interpretation of data in study 1. DBO, LH and CL-Y contributed to conception and design of study 2, DBO contributed to acquisition of data, LH and CL-Y did the analyses and interpretation of data in study 2. LH and CL-Y drafted the manuscript. All authors read and approved the final manuscript.
